# Triple Jeopardy: Complexities of Racism, Sexism, and Ageism on the Experiences of Mental Health Stigma Among Young Canadian Black Women of Caribbean Descent

**DOI:** 10.3389/fsoc.2019.00043

**Published:** 2019-05-15

**Authors:** Dalon Taylor, Donna Richards

**Affiliations:** School of Social Work Department, York University, Toronto, ON, Canada

**Keywords:** Black, race, youth, women, mental health, stigma, Caribbean, intersectionality

## Abstract

This article explores how the intersection of race, gender, and age intertextually complicate and nuance the experience of mental health stigma among young Black women of Caribbean descent living in Canada. The Mental Health Commission of Canada acknowledged that mental health stigma continues to affect the help-seeking behavior of young adults. Some youth-serving agencies and many advocates within Black communities have become increasingly vocal about mental health stigma and the lackluster response to the needs of Black youth (e.g., no increase in funding for the Substance Abuse Program for African, Canadian, and Caribbean Youth—SAPACCY, since the program was established in the mid-1990s). The issue of mental health stigma within the African, Caribbean, and Black Canadian (ACB) communities is widely known and often discussed at public forums. Several recent mental health forums and mental health initiatives held in Toronto made it clear that mental health in Black communities is at a crisis point in the Greater Toronto Area (GTA) and possibly across Canada. Forum discussions also revealed that the issue is further compounded by the intersection of race, gender, and age. In addition, while research studies have also identified stigma as a barrier to accessing mental health services and/or supports, there is a paucity of research on how mental health stigma, when complicated by the experience of racism, sexism, and ageism, affects access to services among young Black women of Caribbean descent. This lack of research on Caribbean women's experience with mental illness limits insights into concepts, issues, and problems that directly impact broader issues related to mental health in Canada. This article engenders a discussion that strengthens the focus on mental health stigma campaigns and education on the mental health of young Black women in Canada. The lack of literature relating to this topic in the Canadian context, as previously noted, limits the extent to which this issue can be fully discussed within Canada. As such, insights into concepts and existing discussions on women's mental health throughout this paper will include references to literature from the U.S., U.K., and Australia, professional experiential knowledge, and personal insights from conversations with young Black women of Caribbean descent. The paper calls for more research on Caribbean women's mental health in Canada to provide better insights and understanding of the issue within a Canadian context.

## Introduction

Perceptions of stigma associated with mental health status are prevalent among racialized populations such as Black youth (Williams and Mohammed, 2009; Khenti, 2013; Black Health Alliance, 2015). The impact of intersecting systems of oppression, such as race, gender, and age on this population, exacerbates their experiences of mental health stigma (Kranke et al., 2012; Jackson-Best, 2017). Due to the historical intersectional locations of these systems of oppression within the axes of marginality, the stigma associated with mental illness poses a critical problem within Black communities (Matthews et al., 2006; Roberts et al., 2008). As depicted in Hollywood movies such as *A Beautiful Mind*, and as documented in scholarly publications, mental illness among White men is often associated with brilliance, while mental illness among Black people is characterized as pathological and an unequivocal sign of weakness (Danquah, 1998). The stigma and oppression caused by the negative perception of mental illness does not bode well for Black families, especially for many Black women who are the backbones of their families. Thus, it is not surprising that there is a disproportionate number of Black youth at the intersection of race and gender who suffer in silence with mental illness and the associated stigma.

This article seeks to examine reports on how the mental health stigma, when it intersects with race and gender, further compounds, complicates, and shapes the mental health experiences of Caribbean and Black female youth of Caribbean descent in Canada. The article engenders a discussion that strengthens the focus on mental health stigma campaigns and education addressing the mental health of young Black women in Canada. To mitigate the paucity of research available in Canada on the topic, insights into concepts and existing discussions on women's mental health throughout this paper will include references to literature from the U.S., U.K., and professional experiential knowledge, and personal insights from conversations with young Black women of Caribbean descent.

For the purpose of this discussion the term *Caribbean* refers to Black individuals born in the Caribbean and living in Canada, as well as individuals born in Canada whose parents are of Caribbean heritage. The terms *young adults* and *youth* are used interchangeably throughout this discussion to refer to young people aged 18–25 years of age (Government of Canada, 2018). The focus on age includes references to youth and young women which is reflective of a given phase on the age continuum. Similarly, focus on the concept of gender which encapsulates either of the sexes, notwithstanding those who are gender neutral, includes reference to the term “female” (and “gender”), and to specific mention of young Black women of Caribbean descent. We also use several ethno-specific terms in our discussion (e.g., Afro-Canadians, African-American, Afro-Caribbean, Black) to refer to Black populations. Black is used as a blanket term to refer to people of African origin or who self-identify as Black. Guided by the lens of intersectionality, this article draws on contemporary examples and on scholarly and gray literature to examine and discuss the high prevalence of mental health stigma in the Canadian Black and Caribbean community, and to discuss the effects of the complexities of race, age, and gender on their experiences with mental illness.

Stigma has been identified as a barrier to accessing mental health services and/or supports for youth populations in general. Various types of stigma that affect the experiences of individuals with mental illness have been identified in discussions on mental health. For example, Clement et al. (2015) outlined six categories of stigma: (1) stigma that is anticipated, whereby the impacted individual expects to be treated or perceived unfairly, (2) stigma that is based on the experience of unfair treatment and unjust perception, (3) stigma that is internalized based on stereotypical views of one's self, (4) perceived stigma by an individual based on the stigmatizing attitudes and behaviors that others direct toward individuals with mental illness, (5) the endorsement of stigma that results from an individual's negative perception toward others with mental illness, and (6) stigma that is present when an individual seeks or receives treatment as a result of mental illness (11–12). Other discussions have made reference to intersectional stigma that occurs when concrete and identifiable social, economic, and political power allows the identification of differences, the construction of stereotypes, the separation of labeled persons into distinct categories, and the disapproval, rejection, exclusion, and discrimination against the persons so characterized (Parker and Aggelton, 2003; Campbell and Deacon, 2006). These different forms of stigma can no doubt further compound the challenges associated with experiences of mental illness, especially for members of groups that are already oppressed.

*Stigma*, for this discussion, is understood as the product “of devaluing, labeling, and stereotyping that are manifested in the loss of status, unfair and unjust treatment” (Goffman, 1963, p. 11). Discussions on stigma that are conceptualized as a “cut into the skin to symbolize the threat or danger of the stigmatized person” (Goffman, 1963, p. 3; Howarth, 2006, p. 442) will also be incorporated throughout this article. Social psychologists often view stigma as a “mark” (Fine and Asch, 1988; Campbell and Deacon, 2006), “separating individuals from one another based on a socially conferred judgement that some persons or groups are tainted as less than” (Pescosolido et al., 2008, p. 431). Furthermore, the social construction of stigma results in social rejection, devaluation, and discrimination (Goffman, 1963; Jones et al., 1985; Crocker et al., 1998). Experiences of stigma have a deleterious effect on the mental health of members of marginalized populations, and this effect is more pronounced among racialized populations such as Black adults and youth (Williams and Mohammed, 2009).

*Double stigma* (Garry, 2005) refers to members of racialized groups who already suffer from the burden of mental illness, combined with prejudice and discrimination because of their group affiliation. This area is also under-researched in Canada, as are the social and structural inequalities that have resulted from stigmatization. Overall, more needs to be done to better understand the resulting challenges of mental illness within Black communities in Canada and to address the added complexities of race, gender, and age on their mental health experiences.

This discussion begins with a brief introduction about the issue of mental health in Canada among racialized populations, with specific example about the connotation of mental health when it comes to Black people. Specific terms or concepts are highlighted in the introduction, to explain the context in which they are used throughout this discussion. An overview of key considerations within an intersectionality framework is provided, followed by a synopsis of Caribbean women in Canada with noted gaps in Canadian literature on mental health concerns in this population. This discussion also includes an overview of mental health in the Black community in Canada as well as insights on what is known about Black young women's health in a Canadian context. The discussion concludes with a summary and highlights areas for future research on the mental health of young Black women from Caribbean descent in Canada.

## Background

Mental health stigma within the Afro-Canadian communities is said to be at crisis proportions (Benjamin, 2002; Khenti, 2013). Yet, relatively little research has been done to date that examines the effects of stigma on Blacks or other racial/ethnic minority populations in Canada. Several research studies (Jamar, 2013; Okeke, 2013; Ward et al., 2013; Montovani et al., 2017) have identified stigma as a barrier to mental health services and/or supports for many people. However, there is a paucity of research that focuses on how mental health stigma, when compounded by the experience of racism, ageism, and sexism, influences access to services among Black people, particularly young Black Caribbean women.

The effects of mental health stigma on the experiences of Black youth, particularly in Toronto, Canada, is often discussed at public forums within the African, Caribbean, and Black Canadian (ACB) communities. Many youth who are affected by mental illness have shared their experiences openly in these forums. The accounts of similar experiences shared by forum attendees (Black Health Alliance, 2015, 2016) living with mental distress have also made it clear that the compounding factors of race, ethnicity, age, and gender are not fully grasped in mainstream services in Canada. As clinicians and practitioners in the social services field, the authors of this article have spearheaded the planning and organizing of several such forums in Toronto, and have participated in many of these discussions. Through this experience, we have become critically aware of the aforementioned mental health crisis that looms within Black communities in the Greater Toronto Area (GTA) (see Khenti, 2013; Black Health Experience in Health Care, 2016; Massaquoi, 2017).

Recently, some youth-serving agencies and many advocates in Black communities in Canada have become increasingly more vocal about the mental health needs of Black youth. For example, recent calls from the Black community have been made to increase the funding and expand the services of the Substance Abuse Program for African, Canadian, and Caribbean Youth (SAPACCY), which is the only program of its kind across Canada and is available at the Center for Addictions and Mental Health (CAMH) in Toronto. Other demands for action to address mental health have been voiced through activities and programs undertaken by the Young Advocates for Youth Agency and through articles published in the popular media (Taylor, 2017) calling for Canada to make Black health a priority.

On June 29, 2015, *HuffPost* carried an online story by Arti Patel about a 33-year-old Black Canadian actress of Caribbean parentage who lives in Toronto and who is secretly battling depression. According to the article, her family members and friends saw her as an aspiring actress with great potential, but she explained that she felt like she was living a double life; her secret life was marred by a “dirtiness” (which is how she described her state of depression) that no amount of makeup could cover. She further described her depression as being so distressing that she transferred her bank account and other possessions to her brother for fear that she would be driven to the point of taking her own life (Patel, 2015).

A similar account of the compounding impact of stigma and mental health on Black youth in Toronto was shared by another individual in a 2016 forum in Toronto (Black Health Alliance, 2016). While this case depicts the experiences of a young Black man who is a survivor of depression and anxiety, the similarities to the case outlined in the *HuffPost* article are concerning. The struggles with suicidal ideations as described and the compounding impact of the stigma that affected the experience of this individual and other similar cases, are indicative of social and systemic failures. There are many other similar accounts of these experiences among Black youth particularly females, who are reported to be battling isolation, depression, social anxiety, low self-esteem, and other signs of mental distress (Black Health Alliance, 2016, p. 4). This looming crisis is exacerbated by the limited services available to Black youth in the Greater Toronto Area, by the lack of culturally appropriate services, and by the structural barriers and longer wait times to receiving treatment after diagnosis (Black Health Alliance, 2015; Black Health Experience in Health Care, 2016). The help-seeking behaviors among the individuals affected are also added factors (Women's Health in Women's Hands (WHIWH), 2003; Veenstra, 2011; Nestel, 2012) and with the limited research available on the topic, a clear understanding of the issue is challenging. Developing a better understanding, demands an approach that attends to the interlocking systems of oppression and the impact on young Black women of Caribbean descent in Canada.

## Intersectionality Lens

Intersectionality is a concept that emerged during the era of “second wave feminism” and highlights the mutually constitutive forces of gender, sex, race, class, and disability (Crenshaw, 1991; Combahee River Collective, 1995; Nash, 2008; Puar, 2012). Intersectionality, applied in this context, considers the impact of race, gender, age, and other social categories on the experience of stigma among young Black women with mental health issues. The intersection of these categories within the different social, cultural, historical, and economic contexts and their impact on the experience of stigma among young Black women are also of concern within the scope of intersectionality. Lorde (1984) argued that women of color are disempowered when aspects of their identity and experiences are examined in isolation from one another. Bowleg (2012) highlighted three key tenets of intersectionality that make it an ideal perspective from which to explore reports of the experiences of young Black women with mental health concerns. These are: (1) “social identities are not independent and unidimensional but multiple and intersecting, (2) individuals from multiple historically oppressed and marginalized groups are the focal and starting point, and (3) multiple social identities at the micro level (gender, race, disability, sexual orientation etc.) intersect with macro level structural factors (racism, poverty, sexism, ageism, ableism) to produce health disparities” (1270). Intersectionality emphasizes the socially constructed nature of gender and other social categories, and takes the wide range of different experiences, identities, and social relations affecting members of different groups in society which will not fit into one category of analysis.

As such, “this framework highlights the complexity that arises when the subject of analysis includes multiple dimensions of social life and analytical categories” (McCall, 2005, p. 72). Intersectionality has become an all-inclusive framework for theorizing injustice. Using intersectionality theory can highlight the salience of disability, race, age, and other social categories that affect both behavior and access to resources. Intersectionality is an important framework for the examination of experiences of mental health concerns among young Black women and the influences of the associated stigma on their help-seeking behavior (Crenshaw, 1991; Collins, 2000, 2015; Shields, 2008). Intersectionality examines how “multiple systems of oppression simultaneously corroborate and subjugate to conceal deliberate, marginalizing ideological maneuvers that define otherness” (Few, 2007, p. 454). Crenshaw (1989, 1991) developed the concept of intersectionality as a tool to convey “the various ways in which race and gender interact to shape the multiple dimensions of Black women's experiences” (Crenshaw, 1991, p. 1244).

Hankivsky and Cormier (2009) posited that intersectionality strives to explain and interpret multiple and intersecting systems of oppression and privilege. For example, Black Caribbean women, and particularly young Black Caribbean women in Canada, may not only experience exclusion from appropriate health-care research but also may be hesitant to accept or seek help for mental health problems for fear of being further stigmatized. It has also been asserted that when young Black women “…do seek healthcare services for their health and mental health, they are often not taken seriously or their complaints are ignored” (Women's Health in Women's Hands (WHIWH), 2007, p. 5). A 2016 report from Women's Health in Women's Hands pointed out that immigrant women's status or country of origin may also play a significant role in their experiences of mental health stigma and subsequent help-seeking processes. The use of an intersectionality lens, then, will serve to disrupt linear thinking that prioritizes any one category of social identity over others. Hankivsky and Cormier (2009) asserted that it provides an understanding of what is created and experienced at the intersection of two or more axes of oppression (for example, race, ethnicity, class, and gender); it is precisely at this basis of intersection that a completely new status is formed, one that is more than simply the sum of its individual parts (Crenshaw, 1991). It is with this intersectionality lens that this discussion on the mental health of Caribbean women in Canada in a broader sense, and more specifically on young Black women of Caribbean descent in Canada, unfolds.

## Overview of Caribbean Women

As of 2007, Canadians of Caribbean origin make up one of the largest non-European ethno-specific groups in Canada (Lindsay, 2007). According to the 2001 National Household Survey, Caribbean people represented more than half a million of the total Canadian population, 69% of whom resided in Ontario (Lindsay, 2007). The Caribbean community in Canada is also said to be relatively young, with 17% of its population ranging from ages 15 to 24 vs. 13% of the overall population. The *Every Woman's Health Matters* report (Massaquoi, 2017) indicated that 67% of young Black women experienced racism when receiving health-care services and 21% reported disengagement from health care as a result. It also indicated that Caribbean women earn $22,824 annually, which is 33% below the poverty line in Canada, and that there are 3,200 more Black women than men in Canada, 60% of whom are under the age of 35 years (12). What is even more glaring is that the top reason that these young women seek care is as a result of psychological distress caused by depression and anxiety (11).

The paucity of research into Caribbean women's experiences with mental illness is reflected in the lack of research available on the topic as it relates to the Caribbean population. A review of literature pertaining to Black women‘s mental health in Canada revealed that very little has been done, especially research into the mental health of young Caribbean women (Curling, 2013; Okeke, 2013). Searches on several online medical health data bases—such as the Cumulative Index of Nursing and Allied Nursing Health Literature (CINAHL), Medline (OVID), Medline (Pub Med), PsyINFO, Sociological and Social Science Abstracts, and the Google Scholar search engine—did not reveal any literature that speaks specifically to the experiences of young Black Caribbean youth who are living in the diaspora and dealing with mental health issues. Hence a significant portion of the discussion in this article is supported by a combination of gray literature, forum reports, and scholarly sources from other countries. This lack of attention to young Black women‘s health in the Canadian context is a concern considering the growing size of both the Black population and the Caribbean communities. The glaring absence of Caribbean women from mental health literature can perhaps be explained by the historical legacy of slavery that remains a present-day reality for Black people.

According to Adkison-Bradley et al. (2009) and Beauboeuf-Lafontant (2007), the White experience is the standard against which Black people are measured, often to the point of exclusion. For example, most mental health research has been conducted with White subjects, limiting its generalizability to racialized populations (Edge, 2008; Himle et al., 2012). Poverty is feminized and racialized and Black women worldwide experience disproportionate poverty (Jones, 2007; Edge, 2013). While most White children are raised with a sense of entitlement, most Black children in North America are raised to anticipate challenges in life because of their race (Adkison-Bradley et al., 2009; Edge and MacKian, 2010).

Additionally, migration to North America is reported to be traumatic for some Black individuals from the Caribbean because it is often their first experience with racial discrimination; an identical experience of such racism is rarely encountered in the country of origin where Blackness is dominant (Adkison-Bradley et al., 2009). The encounter, coupled with the adaptation process, places significant strains on the coping mechanisms of women from the Caribbean, predisposing them to depression and other mental health distress (Edge, 2008; Soto et al., 2011). Additionally, for Black (Caribbean) women who are faced with the dominant construct of being “superwoman” (Collins, 2000; Adkison-Bradley et al., 2009; Woods-Giscombé, 2010; Okeke, 2013), unfeminine, controlling, and independent, characteristics likening them to men, they are inclined to deny the existence of symptoms of depression (Beauboeuf-Lafontant, 2007; Adkison-Bradley et al., 2009; Woods-Giscombé, 2010). Gregory (2006) argued that Black Caribbean women may be further discriminated against in the United States because of their cultural differences from African Americans and mainstream American culture.

The universal discourse of the Black woman's strength may explain why only an estimated 7% of Black women seek treatment (Beauboeuf-Lafontant, 2007). Findings from a U.K. study that examined prevalence and psychosocial risks for perinatal depression among 12 Black Caribbean women revealed some sobering results. The study explored the women‘s models of help-seeking and suggested that, “the way in which help-seeking is conceptualized within health promotion practice may serve to reinforce the invisibility of Black Caribbean women in mainstream mental health services and associated research” (Edge and MacKian, 2010, p. 106). In addition, Jones and Ford (2008) pointed out that the White-dominant, socially constructed identity of the Black woman places Caribbean women at risk of being stereotyped and pathologized when they find the courage to seek treatment. Instead of asking for help, considering personal needs, or speaking out against injustice, the expectation that they will overcome renders Black women silent about emotional distress, which leads to the damage of their self-esteem, personal and intimate relationships, and physical and mental health (Clarke, 2010; Woods-Giscombé, 2010). Black women are also expected to overcome hardship because ancestors who were enslaved endured worse (Beauboeuf-Lafontant, 2007).

Research to date has barely begun to explore the multifaceted forms of oppression that Black women face. The complexities of the effects of mental health stigma and the intersection of race and gender on the experiences of Black Caribbean women in particular, needs more attention in research. As Williams (2001) contended, the epistemological research in mental health with marginalized ethno-racial communities (such as the Caribbean community in Canada) is even more critical given the under-utilization of mental health services by Black people. For Williams (2001), the problem to be explored is not simply the lack of individuals accessing services, but also the structures and processes that prevent members of some groups, such as Black Caribbean young women in Canada, from not only acknowledging but also from seeking and maintaining support for mental health issues. It may require an investigation into how the issue of mental health is addressed in the Black community in Canada in general, to gather a wider scope of this gap.

## Overview of Mental Health in the Black Community in Canada

The Mental Health Commission of Canada (MHCC) (2014) has acknowledged that mental health stigma continues to affect the help-seeking behavior of young adults. The Canadian Mental Health Association (Canadian Mental Health Association, 2017) reports that 28% of individuals aged 20–29 years experience mental illness in a given year and that suicide accounts for 24% of all deaths among youth 15–24 years old and 16% among 25–44 years old. Chai (2017), writing for Toronto Global News reports that more than one in four Canadians are at “high-risk” of mental health issues, but millennials, women, and people with low incomes are the most susceptible. It is also stated that Canadians living in western Canada and in Ontario are the most vulnerable by region (Canadian Mental Health Association, 2017). It is therefore crucial that research in Canada and elsewhere include mental health stigma as it intersects with oppression in the lives of Blacks and other marginalized groups.

According to Anderson et al. (2015), 60% of Blacks in Ontario have increased risk of psychosis (Cantor-Graae and Selten, 2005; see also Bourque et al., 2011; Hansson et al., 2012). According to an Ontario Health Study (OHS) survey (2007) that looked at South Asian, East Asian, Southeast Asian, and Black Ontarians, while mental health concerns are more prevalent among Black people, they are significantly less likely to see a psychiatrist than members of other groups (OICR News, 2016). Ward et al. (2013) note a similar reluctance among Blacks in the United States. Dr. Natasha Browne, a Toronto-based psychologist who often works with Black clients, has said that not only is mental health difficult to discuss in the Black community, it is also quite hidden (Black Health Alliance, 2015). Other studies have corroborated the finding that “Black people” often delay seeking help for mental health challenges (Alvidrez et al., 2008; Masuda et al., 2012; Rosenfield, 2012). On the other hand, evidence from an Morgan et al. (2006) AESOP research report suggested that Black people make an average of six attempts to get help before accessing care—often through adverse pathways. A key area that needs to be investigated in this research is the nature of the barriers that contribute to the delay in seeking service among some Black people and why multiple attempts are being made to access service. The answers to these queries may be quite telling.

A study conducted by the Wellesley Institute in 2005 that examined the role that “Afro-Canadian” status plays in police or ambulance referral to emergency psychiatric services, revealed that Afro-Canadian patients admitted to hospital with psychosis were more likely to have been brought there by police or ambulance compared to members of other groups. It is worth noting that the 2006 AESOP study on the high rates of psychosis among African-Caribbean in the U.K., revealed that African-Caribbean and Black African patients were more likely to be referred to mental health services by the police or via the criminal justice system over Whites (Morgan et al., 2006). These forcible pathways to treatment are believed to be a major contributing factor to Black people's suspicion of the medical and psychiatric systems, a factor that may partly explain why those affected with mental illness delay seeking help (Jarvis et al., 2005). These forceful means to accessing mental health services are not only of grave concern but are also inhumane responses to the mental health needs of vulnerable individuals, especially youth (Daley et al., 2012; Fernando, 2017).

In a 2006 study conducted by Lovell and Shahsiah for the Across Boundaries organization, which is the only gender and ethno-specific mental health serving agency in the Greater Toronto Area, the substance use among youth of color in the Jane and Finch community (a neighborhood considered to be high-risk for youth due to poverty, unemployment, and lack of educational attainment) was examined. Lovell and Shahsiah (2006) interviewed 300 youth, and completed 20 focus groups and 16 group discussions with youth and mental health workers. Results from this study showed that among the social factors “negatively affecting mental health disparities are racism, stigma, intensified feelings of low self-worth, social exclusion and feelings of inadequacy” (9). They further asserted that almost half of all the young women participants indicated that they often felt sad, while only 15% of the young men reported feelings of sadness, and that 30% of young women had suicidal ideations compared to 15% of male participants with similar thoughts (9).

## What Is Known about Young Black Women's Health in the Canadian Context

Black people make up the third largest ethnic population in Canada, yet they remain the most highly stigmatized of all racialized groups (Adjei, 2018; Ontario Human Rights Commission, 2018). The Stephen Lewis (Lewis, 1992) and the most recent report from the Ontario Human Rights Commission, “A Collective Impact: Interim Report on the Injury into Racial Profiling and Racial Discrimination of Black Persons by the Toronto Police Service” (2018), confirm that Black people in Ontario have experienced and continue to experience a unique kind of racism that has not been encountered by any other racialized group. Findings released in 2019 from Justice Michael Tulloch's “Independent Police Oversight Review” (2018) on carding (randomly stopping of individuals by police to gather identification information for intelligence database) revealed that Black and Indigenous people are disproportionately targeted for these checks by Toronto police (Martin, 2019). The report found no evidence that this practice had any effect on the crime rate, yet there has been strong opposition to discontinue this practice by administrators. These oppressive realities are alarming for Black individuals and racialized groups in Canada, and do not foster trusting relationships with police, especially for young individuals with mental illness.

As mentioned earlier, literature on the health concerns and well-being of young Black women is almost non-existent in Canada. What literature there is on the health and well-being of young racialized women does not address the question of how racism influences their physical and mental health (Williams-Morris, 1996). Dougherty (1999) argued that factors impacting the health of young Black women have been largely neglected in research. Guthrie et al. (1997) contended that young racialized women's health concerns are ignored due to societal messages that promote the belief that their health concerns are not valid or important. The researchers pointed out that within Canadian society, their health problems are often dismissed as cultural anomalies. Other studies suggested that the absence of Caribbean women from mental health research is as a result of their ability to cope with adversity, which further contradicts studies already cited that the prevalence of mental health issues in this community is low (Edge, 2008; Edge and MacKian, 2010).

One of the few studies (albeit dated) that sheds some light on young Black women‘s physical and mental health, is a participatory study conducted in 2003 by Women's Health in Women's Hands (WHIWH). A key point to mention is that this organization is the only exclusive women's community health center in Canada and it specializes in providing services to racialized women. The 2003 report indicated that participants identified their main health problems as being caused by “life stress, depression, family violence and eating disorders” (Women's Health in Women's Hands (WHIWH), 2003, p. 18). This research sheds more light on the negative impact of the intersecting sources of oppressions that young Black women face in their everyday encounters both at the individual and community levels (16). A more recent study done by WHIWH in 2017 (see Massaquoi, 2017) indicated that the physical and mental health among young Black women has not improved. These findings made the gaps in what is known about the added complexities of race, age, and sex on these sources of oppression even more obvious.

Another report by the Ontario Health Promotion E-Bulletin (2007) indicated that engaging young Black women and other racialized women in discussions about their own health and well-being was challenging (3). The report attributes this difficulty in engaging with the identified population to racialization/marginalization and a general lack of culturally relevant services. Researchers looking into the experiences of racialized people need to exercise caution to not allow themselves to overemphasize the effects of culture. Focusing on culture as the root cause of issues that racialized and marginalized populations face heightens the risk of deflecting attention from other more insidious systemic and intersectional injustices such as race and racism (Gateri and Richards, 2017). Issues to do with the intersection of mental health stigma with race and gender, for example, also get overlooked. The intersectional approach is critical to any “understanding of which subgroups may be more affected by discriminatory treatment” (Seaton et al., 2010, p. 1372). When it comes to young Black Caribbean women, the intersection of their ethnicity, immigrant status, gender, and age may result in qualitatively different experiences of discrimination and mental health stigma.

The Diagnostic and Statistical Manual of Mental Disorders (DSM- V), a respected medical resource within the biomedical model of health on which diagnoses of mental health issues are based, is a Western, White-dominant construct (Ussher, 2010). Fernando (2017) noted the racist tendencies embedded in the (psychiatric) diagnoses process, in the “color-blindness that often results with Blacks in the UK being diagnosed with schizophrenia more than other groups” (94). Given historical psychiatrization of Blacks, the legacy of slavery, coupled with negative experiences in the health-care system, for example, Black Caribbean individuals in the U.K. reported receiving poor health care due to racism, and many confirmed mistrust and fear of Western-based models of care (Beauboeuf-Lafontant, 2007; Edge and MacKian, 2010). Despite the absence of similar literature of this nature in Canada, this revelation should not be ignored, considering the overrepresentation of Black people in the Canadian criminal justice and child welfare systems, and the underrepresentation of Blacks in academic and political arenas. The lack of representation in these arenas and many other spaces adds credence to the argument that, while the traditional enslavement and ownership of Black people in the West have been abolished, Black communities are still oppressed (Henry and Tator, 2010).

## Conclusion

Research studies into stigma and mental health among ethno-racial populations have demonstrated that stigma exists; however, exploration of its effects is limited (Anglin et al., 2006; Corrigan et al., 2006; Alvidrez et al., 2008; Kranke et al., 2012). Anglin et al. (2006) noted that differences in stigmatizing attitudes toward mental health and help-seeking behavior have received remarkably little attention (857). According to Noteisha Massaquoi (2017), executive director of WHIWH, “the most important factor that shape the health of Black populations are not medical interventions or the personal lifestyle choices we make but rather the socio-economic and political living conditions we experience, like our stigmatized identities” (5). Several research studies (Jamar, 2013; Okeke, 2013; Ward et al., 2013; Montovani et al., 2017) have identified stigma as a barrier to both mental health services and/or supports for many people. However, there is a paucity of research that focuses on how mental health stigma, when compounded by the experience of racism, ageism, and sexism, influences access to services among Black people, particularly young Black Caribbean women.

Canada's international reputation as a multicultural mosaic presents a unique perspective for consideration. Henry and Tator (2010) argued that this myth of multiculturalism perpetuates the ideology that Canada is color blind, an ideology that simultaneously negates the need for racially inclusive research and perpetuates the false notion that racism no longer exists. This notion is contrary to research that suggests that the high prevalence of mental health problems among Caribbean women in Canada (Schreiber et al., 2000; Jackson and Naidoo, 2012) can be attributed to Black communities continued experience of racial oppression (Beauboeuf-Lafontant, 2007; Adkison-Bradley et al., 2009). While the availability of a universal health-care system in Canada has been cause for Canadians to pride themselves on being a “raceless” society, the works of Kumsa et al. (2014), Danquah (1998), and Adjei (2018) have revealed that there is a particular modeling of Blackness that seems to deny agency to Blacks. One consequence of this denial is the insidious effects of anti-Black racism on Black lives that continue to be a harsh reality for Black Canadians. Universal health care in itself cannot fully address the health-care needs of individuals who are struggling with compounding issues embedded in the social context that directly affect their health and treatment.

Therefore, a critical examination of mental health stigma among Black people must acknowledge the interplay of systemic oppressions, such as racism, ageism, and sexism. While many research studies (Williams and Williams-Morris, 2000; Alvidrez et al., 2008; Thornicroft, 2008; Henderson et al., 2012; Shefer et al., 2012) have identified stigma as a barrier to youth accessing mental health services and/or supports, there is a paucity of research that focuses on how the stigma of mental illness affects Black female youth (aged 18–25 years) access to mental health services and/or supports in Canadian society. Furthermore, many researchers have argued that studies that have been conducted on the stigma associated with mental illness rarely consider the effects and the interplay of race, gender, and other social categories on mental health and access to services (Beauboeuf-Lafontant, 2007; Okeke, 2013; Jones and Guy-Sheftall, 2015; Etowa et al., 2018).

Haraway (1988) entreated researchers and educators to put people's situated knowledge into context when analyzing their lived experiences. In other words, how do Blacks come to experience stigma in the light of social narratives of identity, subjectivity, self, group, and history? Collins (2000) noted that “Black women have been assaulted with a variety of negative images [such as] stereotypical mammies, matriarchs, welfare recipients, and hot mommas, to help justify Black women's oppression” (69). Given the prevalence of such images, mental health stigma, when intersected with other powerful identity markers such as race, gender, and age may have unique effects on young Black women. As there has been little research done specifically with young Black women in Canada and even less on Black female youth and mental health stigma at the intersection of race, gender, and ageism, more research is needed on this population. Even more needs to be done to address the issue as it relates to young Black women of Caribbean descent in Canada.

The domains of stigmatization are multifaceted in how they affect people's lives. Stigmatization can have dramatic effects on the distribution of life chances in areas such as earnings, housing, criminality, health, mental health, and life itself (Anglin et al., 2006). Several scholars whose work focuses on stigma have linked stigmatized identities to psychological distress, while others urge further research into how psychological identity working in tandem with stigma affects a person's vulnerability to distress (Quinn and Chaudoir, 2009). With this in mind, it is clear that health-care providers need to increase efforts to address these glaring gaps in service as well.

The absence of dialogue about Caribbean women's experience with mental health, the management of mental health concerns and the extremely limited research on these issues are notable both within and outside the Caribbean community. It is critical that health and mental health professionals acknowledge and validate the ways in which Caribbean women experience mental illness and treatment (Waldron, 2010; Okeke, 2013; Etowa et al., 2018). It is even more crucial that their experiences be “understood and addressed within the socio-cultural, historical, and political context of their lives” (O‘Mahony and Donnelly, 2010, p. 440). An intersectional approach is therefore warranted not only in future research with this population but also in practice, given that they continue to experience the “matrix of intersectional oppressions” (Collins, 2000). Addressing these areas are crucial for a country such as Canada to demonstrate true inclusivity in research on mental health.

## Author Contributions

DT and DR debated the content and organization of this paper and both contributed equally to the final production of the manuscript.

### Conflict of Interest Statement

The authors declare that the research was conducted in the absence of any commercial or financial relationships that could be construed as a potential conflict of interest.

## References

[B1] Adjei P. B. (2018). The (em)bodiment of blackness in a visceral and anti-black racism and ableism context. Race Ethnicity Educ. 21, 275–287. 10.1080/13613324.2016.1248821

[B2] Adkison-Bradley C. Maynard D.-M. Johnson P. Carter S. (2009). British African Caribbean women and depression. Br. J. Guid. Counc. 37, 67–71. 10.1080/03069880802535887

[B3] Alvidrez J. Snowden L. R. Kaiser D. (2008). The experience of stigma among Black mental health consumers. J. Health Care Poor Undeserved. 19, 874–893. 10.1353/hpu.0.0058 18677076

[B4] Anderson K. K. Cheng J. Susser E. McKenzie K. Kurdyak P. (2015). Incidence of psychotic disorders among first-generation immigrants and refugees in Ontario. Can. Med. Assoc. J. 187, E279–E286. 10.1503/cmaj.141420 25964387 PMC4467956

[B5] Anglin D. Link B. Phelan J. (2006). Racial differences in stigmatizing attitudes toward people with mental illness. Psychiatr. Serv. 57, 857–862. 10.1176/ps.2006.57.6.857 16754764

[B6] Beauboeuf-Lafontant T. (2007). You have to show strength: an exploration of gender, race and depression. Gender Soc. 21H, 28–51. 10.1177/0891243206294108

[B7] Benjamin A. (2002). The social and legal banishment of anti-racism: A Black perspective, in Crimes of Colour: Racialization and the Criminal Justice System in Canada, eds Chan W. Mirchandi K. (Peterborough, ON: Broadview Press), 177–190.

[B8] Black Health Alliance (2015). A Sound Mind: Mental Health in the Black Community, Forum Report. Toronto, ON: Black Health Alliance.

[B9] Black Health Alliance (2016). A Sound Mind II: Mental Health in the Black Community, Forum Report. Toronto, ON: Black Health Alliance.

[B10] Black Health Experience in Health Care (2016). Health and Mental Health in the Black Community, Symposium Report. Toronto, ON: Sinai Health Systems on Human Rights and Health Equity Office.

[B11] Bourque F. van der Ven E. Malla A. (2011). A meta-analysis of the risk for psychotic disorders among first and second-generation immigrants. Psychol. Med. 41, 897–910. 10.1017/S0033291710001406 20663257

[B12] Bowleg L. (2012). The problem with the phrase women and minorities: intersectionality – an important framework for public health. Am. J. Public Health 102, 1267–1273. 10.2105/AJPH.2012.300750 22594719 PMC3477987

[B13] Campbell C. Deacon H. (2006). Unravelling the contexts of stigma: from internalisation to resistance to change. J. Commun. Appl. Soc. Psychol. 16, 411–417. 10.1002/casp.901

[B14] Canadian Mental Health Association (2017). Mental Health Statistics. Toronto, ON: Canadian Mental Health Association (CMHA).

[B15] Cantor-Graae E. Selten J.-P. (2005). Schizophrenia and migration: a meta-analysis and review. Am. J. Psychiatry 162, 12–24. 10.1176/appi.ajp.162.1.12 15625195

[B16] Chai C. (2017). Why more Canadians millennials than ever are at “high risk” of mental health issues. Toronto Global News.

[B17] Clarke J. (2010). The portrayal of depression in the three most popular English language Black-American magazines in the USA: Ebony, Essence, and Jet. Ethnicity Health 15, 459–473. 10.1080/13557858.2010.488261 20818573

[B18] Clement S. Schauman O. Graham T. Maggioni F. (2015). What is the impact of mental health-related stigma on help-seeking? A systematic review of quantitative and qualitative studies. Psychol. Med. 45, 11–27. 10.1017/S0033291714000129 24569086

[B19] Collins P. H. (2000). Black Feminist Thought: Knowledge, Consciousness, and the Politics of Empowerment, 2nd Edn. New York, NY: Routledge.

[B20] Collins P. H. (2015). Intersectionality's definition dilemmas. Annu. Rev. Sociol. 41, 1–20. 10.1146/annurev-soc-073014-112142

[B21] Combahee River Collective (1995). A black feminist statement, in Words of Fire: An Anthology of African American Feminist Thought, ed Guy-Sheftall B. (New York, NY: New Press), 232–240.

[B22] Corrigan P. W. Watson A. C. Barr L. (2006). The self-stigma of mental illness: implications for self-esteem and self-efficacy. J. Soc. Clin. Psychol. 25, 875–884. 10.1521/jscp.2006.25.8.875

[B23] Crenshaw K. (1989). Demarginalizing the intersection of race and sex: a Black feminist critique of discrimination doctrine, feminist theory and politics. Univ. Chicago Legal Forum 8, 139–167.

[B24] Crenshaw K. (1991). Mapping the margins: intersectionality, identity politics, and violence against women of colour. Stanford Law Rev. 43, 1241–1299. 10.2307/1229039

[B25] Crocker J. Major B. Steeles C. M. (1998). Social stigma and self-esteem: The self protective properties of stigma. Psychol. Rev. 96, 608–630. 10.1037/0033-295X.96.4.608

[B26] Curling D. (2013). I Heal, We Heal: A Qualitative Study of Black Canadian Women‘s Experiences of Depression and Coping (Theses/Dissertation).1–269.

[B27] Daley A. Costa L. Ross L. (2012). (W)righting women: constructions of gender, sexuality and race in the psychiatric chart. Cult. Health Sexual. 14, 955–969. 10.1080/13691058.2012.712718 22900575

[B28] Danquah M. N.-A. (1998). Willow Weep for Me: A Black Woman's Journey through Depression. New York, NY: Ballantine.

[B29] Dougherty D. M. (1999). Health care for adolescent girls, in Beyond Appearance: A New Look at Adolescent Girls, eds Johnson N. G. Roberts M. C. Worell J. (Washington, DC: American Psychological Association Press), 301–325.

[B30] Edge D. (2008). We don't see Black women here: an exploration of the absence of Black Caribbean women from clinical and epidemiological data on perinatal depression in the UK. Midwifery 24, 379–389. 10.1016/j.midw.2007.01.007 17714839

[B31] Edge D. (2013). Why are you cast down, o my soul? Exploring the intersections of ethnicity, gender, depression, spirituality and implications for Black Caribbean women‘s mental health. Crit. Public Health 23, 39–48. 10.1080/09581596.2012.760727

[B32] Edge D. MacKian S. (2010). Ethnicity and mental health encounters in primary care: help-seeking and help-giving for prenatal depression among Black Caribbean women in the UK. Ethnicity Health 15, 93–111. 10.1080/13557850903418836 20077242

[B33] Etowa J. Keddy I. Egbeyemi J. Eghan F. (2018). Depression: the “invisible fog” influencing the midlife health of African Canadian women. Int. J. Ment. Health Nurs. 16, 203–213. 10.1111/j.1447-0349.2007.00469.x 17535166

[B34] Fernando S. (2017). Institutional Racism in Psychiatry and Clinical Psychology: Race Matters in Mental Health. London: Palgrave Macmillan.

[B35] Few A. (2007). Integrating Black consciousness and critical race feminism into family studies research. J. Fam. Issues 28, 452–473. 10.1177/0192513X06297330

[B36] Fine M. Asch A. (1988). Disability beyond stigma: social interaction, discrimination and activism. J. Soc. Issues 44, 3–21. 10.1111/j.1540-4560.1988.tb02045.x

[B37] Garry F. A. (2005). Stigma: Barrier to mental health-care among ethnic communities. Issues Ment. Health Nurs. 26, 979-999. 10.1080/01612840500280638 16283995

[B38] Gateri H. Richards D. (2017). Exploring barriers refugees and refugee claimants experience accessing reproductive health care in Toronto. J. Refugee Rev. 3, 122–136.

[B39] Goffman E. (1963). Stigma: Notes on the Management of Spoiled Identity. Englewood Cliffs, NJ: Prentice Hall.

[B40] Government of Canada (2018). A portrait of Canadian youth- Statistics Canada. Available online at: https://www.150.statcan.gc.ca/n1/pub11-631-x2018001-eng.htm (Retrieved August 15, 2018).

[B41] Gregory S. T. (2006). The cultural constructs of race, gender and class: a study of how Afro-Caribbean women academics negotiate their careers. Int. J. Qual. Stud. Educ. 19, 347–366. 10.1080/09518390600696877

[B42] Guthrie B. J. Caldwell H. Hunter A. G. (1997). Minority adolescent female health, in Health-Promoting and Health-Compromising Behaviors Among Minority Adolescents, eds Wilson D. K. Rodrigue J. R. Taylor W. C. (Washington, DC: American Psychological Association Press), 153–171.

[B43] Hankivsky O. Cormier R. (2009). Intersectionality: Moving Women‘s Health Research Policy Forward. Vancouver, BC: Women's Health Research Network.

[B44] Hansson E. K. Tuck A. Lurie S. McKenzie K. (2012). Rates of mental illness and suicidality in immigrant, refugee, ethnocultural, and racialized groups in Canada: a review of the literature. Can. J. Psychiatry 57, 111–121. 10.1177/070674371205700208 22340151

[B45] Haraway D. (1988). Situated knowledges: the science question in feminism and the privilege of partial perspective. Fem. Stud. 14, 575–599. 10.2307/3178066

[B46] Henderson C. Evans-Lacko S. Flach C. Thornicroft G. (2012). Responses to mental health stigma questions: the importance of social desirability and data collection method. Can. J. Psychiatry 57, 152–160. 10.1177/070674371205700304 22398001

[B47] Henry F. Tator C. (2010). The Colour of Democracy: Racism in Canadian Society, 4th Edn. Toronto, ON: Nelson Education.

[B48] Himle J. Taylor R. Chatters L. (2012). Religious involvement and the obsessive compulsive disorder among African Americans and Black Caribbeans. J. Anxiety Disord. 26, 502–510. 10.1016/j.janxdis.2012.02.003 22397898 PMC11610501

[B49] Howarth C. (2006). Race as stigma: positioning the stigmatized as agents, not objects. J. Commun. Appl. Soc. Psychol. 16, 442–451. 10.1002/casp.898

[B50] Jackson F. Naidoo K. (2012). “Lemeh check see if meh mask on straight”: examining how Black women of Caribbean descent in Canada manage their depression and construct womanhood through being strong. Southern J. Can. Stud. 5, 223–240. 10.222.15/sjes.vSil.296

[B51] Jackson-Best F. (2017). Researching Women's Mental Health. Muslink, Canada Online Hub (accessed February 13, 2017).

[B52] Jamar R. S. (2013). Stigma, Race and Mental Illness: African American Clinicians' Perceptions of How These Factors Influence Help-Seeking Behaviors in African Americans (Theses, Dissertations and pRojects), 1–73. Available online at: https://scholarworks.smith.edu/theses/568 (Retrieved August 17, 2018).

[B53] Jarvis G. Kirmayer L. Jarvis G. K. Whiteley R. (2005). The role of Afro-Canadian status in police or ambulance referral to emergency psychiatrist services. Psychiatr. Serv. 56, 705–710. 10.1176/appi.ps.56.6.705 15939947

[B54] Jones E. Farina A. Hastorf A. Markus A. Miller D. Scott R. (1985). Social Stigma: The Psychology of Marked Relationships. New York, NY: Freeman.

[B55] Jones L. (2007). Preventing depression: culturally relevant work with Black women. Res. Soc. Work Pract. 18, 626–634. 10.1177/1049731507308982

[B56] Jones L. Ford B. (2008). Depression in African American women: application of a psychosocial competence practice framework. J. Women Soc. Work 23, 134–143. 10.1177/0886109908314324

[B57] Jones L. V. Guy-Sheftall B. (2015). Conquering the Black girl blues. Soc. Work 60, 343–350. 10.1093/sw/swv032 26489355

[B58] Khenti A. (2013). Homicide among young Black men in Toronto: an unrecognizable public health crisis? Can. J. Public Health 104, e12–e14. 10.2105/AJPH.2011.300264e 23618113 PMC6974223

[B59] Kranke D. Guada J. Kranke B. Floersch J. (2012). What do African American youth with mental illness think about help-seeking and psychiatric medication? Origins of stigmatizing attitudes. Soc. Work Ment. Health 10, 53–71. 10.1080/15332985.2011.618076

[B60] Kumsa M. K. Mfoafo-Mc‘Carthy M. Oba F. Gaasim S. (2014). The contours of Anti-Black Racism: engaging anti-oppression from embodied spaces. J. Crit. Anti Oppressive Soc. Inquiry 1, 21–38.

[B61] Lewis S. (1992). Report of the Advisor on Race Relations to the Premier of Ontario. Bob Rae. Report.

[B62] Lindsay C. (2007). “Profiles of Ethnic Communities in Canada”: The Caribbean Community in Canada. Ottawa, ON: S.C. Catalogue.

[B63] Lorde A. (ed.). (1984). Sister Outsider- Essays and Speeches. New York, NY: Ten Speed Press.

[B64] Lovell A. Shahsiah S. (2006). Mental Well-Being and Substance Use among Youth of Colour. Toronto, ON: Across Boundaries.

[B65] Martin T. (2019). Judge‘s call to ban carding now rests with Ontario Government. The Canadian Press. Available online at: https://www.theglobeandmail.com/canada/article-judges-call-to-ban-carding-now-rests-with-ontario-government/ (Retrieved September 24, 2018).

[B66] Massaquoi N. (2017). Every Woman's Health Matters: Developing a Data-Informed Practice to Reduce Health Disparities for Black Women Accessing Healthcare in Toronto. Toronto, ON: Women's Health in Women‘s Hands.

[B67] Masuda A. Anderson P. L. Edmonds J. (2012). Help-seeking attitudes, mental health stigma, and self-concealment among African American College students. Psychol. Faul. Publ. 91, 1–20. 10.1177/0021934712445806

[B68] Matthews A. K. Corrigan P. W. Smith B. M. Aranda F. (2006). A qualitative exploration of African-Americans' attitudes toward mental illness and mental illness treatment seeking. Rehabil. Educ. 20, 253–268. 10.1891/088970106805065331

[B69] McCall L. (2005). The complexity of intersectionality. J. Women Cult. Soc. 30, 1771–1800. 10.1086/426800

[B70] Mental Health Commission of Canada (MHCC) (2014). Headstrong. Available online at: http://www.mentalhealthcommission.ca/English/initiatives/11876/headstrong (Retrieved September 7, 2018).

[B71] Montovani N. M Pizzolati Edge D. (2017). Exploring the relationship between stigma and help-seeking for mental illness in African-descended faith communities in the UK. Health Expect. 20, 373–384. 10.1111/hex.12464 27124178 PMC5433535

[B72] Morgan C. Dazzan P. Morgan K. Jones P. Harrison G. Leff J.. (2006). First episode psychosis and ethnicity: initial findings from the AESOP study. World Psychiatry 5, 40–46. 10.1192/bjp.106.021303 16757995 PMC1472260

[B73] Nash J. C. (2008). Rethinking intersectionality. Fem. Rev.. 89, 1–15. 10.1057/fr.2008.4

[B74] Nestel S. (2012). Colour Coded Health Care: The Impact of Race and Racism on Canadian Health. Toronto, ON: The Wellesley Institute.

[B75] O‘Mahony J. Donnelly T. (2010). A postcolinial feminist perspective inquiry into immigrant women‘s mental health and experiences. Issues Ment. Health Nurs. 31, 440–449. 10.3109/01612840903521971 20521913

[B76] OICR News (2016). Study Examines Mental Health in Common Ethnic Minorities in Ontario. Available online at: https://news.oicr.on.ca/2016/06/study-examines-mental-health-in-common-ethnic-minorities-in-ontario/ (Retrieved September 7, 2018).

[B77] Okeke A. (2013). A Culture of Stigma: Black Women and Mental Health. Undergraduate Research Paper Awards. Available online at: http://scholarworks.gsu.edu/univ_lib_ura/13 (Retrieved September 24, 2018).

[B78] Ontario Human Rights Commission (2018). A Collective Impact Report on the Inquiry into Racial Profiling and Racial Discrimination of Black Persons by Toronto Police Service, Interim Report. Toronto, ON: Otario Human Rights Commission (OHRC).

[B79] Parker R. Aggelton P. (2003). HIV and AIDS-related stigma and discrimination: A conceptual; framework and implications for action. Soc. Sc. Med. 57, 13–24. 10.1016/S0277-9536(02)00304-0 12753813

[B80] Patel A. (2015). Stigma and silence: Black Canadians and the fight for mental health awareness. Toronto Global News. Available online at: https://globalnews.ca/author/arti-patel (Retrieved July 16, 2018).

[B81] Pescosolido B. A. Martin J. K. Lang A. Olafsdottir S. (2008). Rethinking theoretical approaches to stigma: a framework integrating normative influences on stigma (FNIS). Soc. Sci. Med. 67, 959–969. 10.1016/j.socscimed.2008.03.018 PMC2587424 18436358

[B82] Puar J. K. (2012). I would rather be a cyborg than a goddess: Becoming-intersectional in assemblage theory. J. Fem. Philo. 2, 49–66.

[B83] Quinn D. M. Chaudoir S. R. (2009). Living with a concealable stigmatized identity: the impact of anticipated stigma, centrality, salience, and cultural stigma on psychological distress and health. J. Pers. Soc. Psychol. 97, 634–651. 10.1037/a0015815 19785483 PMC4511710

[B84] Roberts K. T. Robinso K. M. Top R. Newman J. Smith F. Stewart C. (2008). Community perceptions of mental health needs in an undeserved minority neighbourhood. J. Commun. Health Nurs. 25, 2013–2017. 10.1080/07370010802421202 18979331

[B85] Rosenfield S. (2012). Triple jeopardy? Mental health and the intersection of gender, race, and class. Soc. Sci. Med. 74, 1791–1801. 10.1016/j.socscimed.2011.11.010 22326306

[B86] Schreiber R. Noerager Stern R. Wilson P. (2000). Being strong: how Black West-Indian Canadian women manage depression and its stigma. J. Nurs. Scholarsh. 32, 39–45. 10.1111/j.1547-5069.2000.00039.x 10819737

[B87] Seaton E. K. Caldwell C. H. Sellers R. M. Jackson J. S. (2010). An intersectional approach for understanding perceived discrimination and psychological well-being among African American and Caribbean Black youth. Dev. Psychol. 46, 1372–1379. 10.1037/a0019869 20822246 PMC3755457

[B88] Shefer G. Rose D. Nellums L. Thornicroft G. Henderson C. S. Evans-Lacko (2012). “Our community is the worst”: the influence of cultural beliefs on stigma, relationships with family and help-seeking in three ethnic communities in London. Int. J. Soc. Psychiatry. 59, 535–544. 10.1177/0020764012443759 22588248

[B89] Shields S. A. (2008). Gender: an intersectional perspective. Sex Roles 59, 301–311. 10.1007/s11199-008-9501-8

[B90] Soto J. Dawson-Andoh N. Belue R. (2011). The relationship between perceived discrimination and generalized anxiety disorder among African Americans, Afro Caribbeans, and non-Hispanic Whites. J. Anxiety Disord. 25, 258–265. 10.1016/j.janxdis.2010.09.011 21041059 PMC3053120

[B91] Taylor D. P. (2017). Black health needs to become a priority. The Toronto Star.

[B92] Thornicroft G. (2008). Stigma and discrimination limit access to mental health care. Epidemiol.Psichiatr. Soc. 17, 14–19. 10.1017/S1121189X00002621 18444452

[B93] Ussher J. (2010). Are we medicalizing women‘s misery? A critical review of women‘s higher rates of reported depression. Fem. Psychol. 20, 9–35. 10.1177/0959353509350213

[B94] Veenstra G. (2011). Race, gender, class, and sexual orientation: Intersecting axes of inequality and self-rated health in Canada. Int. J. Equity Health 10, 1–10. 10.1186/1475-9276-10-3 21241506 PMC3032690

[B95] Waldron I. (2010). The marginalization of African indigenous healing traditions within Western medicine: reconciling ideological tensions and contradictions along the epistemological; terrain. Womens Health Urban Life 9, 50–68. Available online at: http://hdl.handle.net/1807/24423

[B96] Ward E. C. Wilshire J. C. Detry M. A. Brown R. L. (2013). African American men and women's attitude toward mental illness, perceptions of stigma, and preferred coping behaviors. Nurs. Res. 62, 185–194. 10.1097/NNR.0b013e31827bf533 23328705 PMC4279858

[B97] Williams C. (2001). Increasing access and building equity into mental health services: an examination of potential for change. Can. J. Commun. Ment. Health 20, 37–51. 10.7870/cjcmh-2001-0003 11599135

[B98] Williams D. R. Mohammed S. A. (2009). Discrimination and racial disparities in health: evidence and needed research. J. Behav. Med. 32, 20–47. 10.1007/s10865-008-9185-0 19030981 PMC2821669

[B99] Williams D. R. Williams-Morris R. (2000). Racism and mental health: the African American experience. Ethnicity Health 3, 243–268. 10.1080/713667453 11105267

[B100] Williams-Morris R. S. (1996). Racism and children's health. Ethnicity Dis. 6, 69–82. 8882837

[B101] Women's Health in Women's Hands (WHIWH) (2007). The Challenges of Engaging Young Black Women and Women of Colour in Healthcare Service Delivery Systems, Including Community Health Centres. Ontario Health Promotion E-Bulletin. Available online at: https://www.ohpe.ca/node/10437 (Retrieved September 7, 2018).

[B102] Women's Health in Women's Hands (WHIWH) (2003). Racial Discrimination as a Health Risk for Female Youth: Implications for Policy and Healthcare Delivery in Canada. Toronto, ON: Canadian Race Relations Foundation. Available online at: http://accessalliance.ca/wp-content/uploads/2015/03/ePubRepRacDiscYouth.pdf (Retrieved July 15, 2018).

[B103] Woods-Giscombé C. L. (2010). Superwoman schema: African American women's views on stress, strength, and health. Qual. Health Res. 20, 668–683. 10.1177/1049732310361892 20154298 PMC3072704

